# Biliary-enteric reconstruction in laparoscopic radical resection of hilar cholangiocarcinoma: a single-center retrospective cohort study

**DOI:** 10.1186/s12885-023-10942-y

**Published:** 2023-05-18

**Authors:** Wenzheng Liu, Fei Xiong, Guanhua Wu, Qi Wang, Bing Wang, Yongjun Chen

**Affiliations:** Department of Biliary-Pancreatic Surgery, Tongji Hospital, Tongji Medical College, Huazhong University of Science and Technology, 1095 Jiefang Avenue, Wuhan, 430030 Hubei China

**Keywords:** Laparoscopic radical resection of hilar cholangiocarcinoma, Biliary-enteric anastomosis

## Abstract

**Objective:**

To evaluate the feasibility and quality of biliary-enteric reconstruction (BER) in laparoscopic radical resection of hilar cholangiocarcinoma (LsRRH) versus open surgery and propose technical recommendations.

**Methods:**

Data of 38 LsRRH and 54 radical laparotomy resections of hilar cholangiocarcinoma (LtRRH) cases were collected from our institution. BER was evaluated via biliary residuals numbers, number of anastomoses, anastomosis manner, suture method, time consumption, and postoperative complication.

**Results:**

In the LsRRH group, patients were relatively younger; Bismuth type I had a higher proportion while type IIIa and IV were less and required no revascularization. In LsRRH and LtRRH groups, respectively, the biliary residuals number was 2.54 ± 1.62 and 2.47 ± 1.46 (p > 0.05); the number of anastomoses was 2.04 ± 1.27 and 2.57 ± 1.33 (p > 0.05); the time of BER was 65.67 ± 21.53 and 42.5 ± 19.77 min (p < 0.05), 15.08 ± 3.64% and 11.76 ± 2.54% of the total operation time (p < 0.05); postoperative bile leakage incidence was 15.79% and 16.67% (p > 0.05); 14 ± 10.28 and 17 ± 9.73 days for healing (p < 0.05); anastomosis stenosis rate was 2.63% and 1.85% (p > 0.05). Neither group had a biliary hemorrhage or bile leakage-related death.

**Conclusion:**

The selection bias in LsRRH mainly affects tumor resection than BER. Our cohort study indicates that BER in LsRRH is technically feasible and equals anastomotic quality to open surgery. However, its longer and a more significant proportion of total operation time represent that BER has higher technical requirements and is one of the critical rate-limiting steps affecting the minimal invasiveness of LsRRH.

Hilar Cholangiocarcinoma (HCCA) has been considered the most challenging tumor since it was first described by Altemeier in 1957 [1] and popularized by Klatskin in 1965 [2] because of its strong invasiveness and poor prognosis [3]. Radical resection of HCCA [4] is an important basis for radical treatment and improved prognosis. Surgical treatment combined with chemoradiotherapy or the emerging targeted immunotherapy [5] has made tremendous progress over the past 50 years, with a 5-year disease-specific survival rate of about 40% and a median disease-specific survival rate of more than 40 months [6].

Although the surgical strategy of HCCA with different Bismuth-Corllete classification and TNM stageing is controversial, surgical methods usually consist of hilar dissection of lymphatic-adipose-nerve tissues, combined hepatectomy with total caudal lobectomy, extrahepatic bile duct resection and biliary-enteric reconstruction (BER) [7], and vascular resection and reconstruction in necessary cases [8]. Radical resection of HCCA is noticeably complex and traumatic, and patients have to suffer significant surgical and anesthetic trauma as well as potential complications.

Laparoscopic techniques have been successful in almost all abdominal operations [9–11]. Promising results from the inherent minimal invasiveness of laparoscopic surgery are frequently reported, including minor intraoperative trauma, fewer postoperative complications, and enhanced recovery after surgery (ERAS), which attracts hepatobiliary surgeons to explore laparoscopic radical resection of hilar cholangiocarcinoma (LsRRH). However, the exceptionally high technique requirements and concerns about oncological ineffectiveness have limited the adoption of LsRRH [12].

More than 20 institutions have published their experience claiming the technical safety, radicality, and feasibility of LsRRH performed by experienced surgeons on carefully selected patients in high-volume hepatobiliary centers [13–17]. But the minimal invasiveness of LsRRH remains controversial [12, 18, 19]. Laparoscopic BER is an important rate-limiting step in LsRRH. However, there are no monographic clinical studies on the advantage and disadvantages of BER in LsRRH and the effect of BER on the feasibility and minimally invasive of LsRRH. In response to these concerns, we performed a comparative study of BER, focusing on its time percentage and technical complexity in LsRRH and LtRRH, and the impact of BER on postoperative complications and immediate and long-term outcomes of the overall operation.

## Methods

### Patients’ cohort and data collection

Data of 92 HCCA patients in our institution between January 2017 and December 2020 were collected and divided into LsRRH (n = 38) and LtRRH (n = 54) groups according to the planned surgical procedure preoperatively. Three patients in the LsRRH group who were converted to LtRRH were excluded from the statistics. All three patients were found to be laparoscopically inaccessible intraoperatively due to the anatomical location of the tumor, which was an inadequate preoperative assessment and not related to the LsRRH. All patients were pathologically proven as HCCA.

Data was recorded by a standard case report form, which contained Bismuth-Corlette classification, preoperative biochemical parameter, operation time, blood loss and transfusion, margin status (R0/R1/R2), postoperative complications (according to the Clavien-Dindo classification [20]), drainage duration, postoperative length of hospitalization, and 30-days mortality. Data related to BER were recorded in detail to meet this study’s purpose. Emphasis was on the number of biliary residuals and number of anastomoses, anastomosis manner, suture method, and time consumption. Postoperative complications mainly focused on bile leakage, anastomotic stenosis, and BER-related death.

### Criteria of patients selected for LsRRH

Currently, there are no international criteria for LsRRH except an expert consensus on LRRHcca standards from The Expert Group on Operational Norms of Laparoscopic Radical Resection of Perihilar Cholangiocarcinoma [21]. Patients enrolled for LsRRH in our institution comply with three principles. The first is to meet the resectability and radicality requirements of LtRRH as described in previous studies [22, 23]. Concretely, HCCA with distant or intrahepatic metastasis, peritoneal seeding, and para-aortic lymph node metastasis is considered unresectable. The second is the absence of hepatic artery (HA) or/and portal (PV) invasion, which means no vascular resection and reconstruction are required. Finally, the patient can tolerate pneumoperitoneum and prolonged anesthesia.

### Preoperative management

All patients underwent B-ultrasound, contrast-enhanced CT, and magnetic resonance cholangiopancreatography (MRCP) to collect for 3-D imaging. The tumor size, location, extent of bile duct invasion, Bismuth-Corlette classification, lymph nodes, distant metastasis, HA and PV involvement, and tumor stage were then assessed. The preoperative plan carefully evaluated the prospective number, technique, and location of BER.

Endoscopic retrograde cholangiopancreatography (ERCP) and magnetic resonance plain scan with perfusion and diffusion imaging was not routinely conducted in patients with ambiguous diagnoses, especially in distinguishing sclerosing and infiltrating HCCA tumors from cholangitis.

Patients with total bilirubin (TB) above 100µmol/L received preoperative biliary drainage. Patients first diagnosed in our center received external drainage using preferentially percutaneous transhepatic biliary drainage (PTCD). Patients referred to our center who already had biliary drainage would maintain their existing protocol. Even if the pre-preserved liver has been determined in the preoperative surgery plan, we recommended bilateral PTCD to adapt to the possible change of hepatectomy during intraoperative exploration.

To avoid the impact of cholangitis on the outcome of the surgery, for patients with cholangitis, we use PTCD or other drainage methods until the bile is clear, the patient has no abdominal pain or fever.

To mitigate nutritional and immune damage caused by bile loss, a jejunal nutrient tube was placed in the duodenum by duodenoscope for bile retrieval. Surgery would be deferred for 2 to 3 weeks until TB was reduced to less than 100 µmol/L. An indocyanine green (ICG) clearance test was performed for patients undergoing major hepatectomy when TB < 100 µmol/L to assess the future liver remnant (FLR) as well as to ensure at least 40% was preserved.

### Postoperative management

Postoperative complications were totalized according to the definition and grade of bile leakage by the International Study Group of Liver Surgery (2011) [24]. The criterion for healing bile leakage is no abdominal fluid collection, and a drainage tube can be removed. Those who cannot remove the drainage tube with continuous bile drainage should be kept for 2 ~ 3 months until the sinus tract forms firmly and clamped the tube for seven days. Then the drainage tube can be removed after the patient has no effusion identified by CT and no abdominal pain or fever.

### Statistical analysis

Normally distributed continuous variables are presented as means ± standard deviation (SD) and analyzed using Student’s t-test for independent samples. Continuous variables not following normal distribution are presented as medians with interquartile range (IQR) or content and were analyzed using the Mann-Whitney U test. As appropriate, categorical variables are presented as numbers and percentages and analyzed using Chi-square or Fisher’s exact test. P < 0.05 was considered statistically significant, and all statistical analyses were conducted using SPSS® version 24 (IBM Corp., Armonk, NY, USA).

## Results

### Demographic and baseline before surgery

Details of the demographics and baseline before surgery are summarized in Table 1. There was no difference between LsRRH and LtRRH groups in gender, BMI, total and direct bilirubin, and preoperative biliary drainage manner. However, the mean age of patients in the LsRRH group was significantly younger than the LtRRH group (P < 0.05), suggesting that patients with better condition and tolerance for pneumoperitoneum and prolonged anesthesia were selected for laparoscopic surgery. All patients with T-Bil > 100µmol/L underwent PTBD except 6 patients who underwent ENBD in referral hospitals. Eight cases with T-Bil < 100µmol/L in both groups did not undergo biliary drainage. A total of 5 patients in both groups did not experience bile reinfusion due to the resistance of the jejunal nutrient tube implement. No difference in mean biliary drainage time and ICG_15_% between the two groups were observed.

**Table 1:** Demographics and baseline

	LsRRH (n = 38)	LtRRH (n = 54)	*p*Value
Gender (M/F)	20/18	25/29	
Age	56.70 ± 12.31	64.22 ± 10.47	< 0.05
BMI	27.61 ± 3.48	26.39 ± 2.79	
T-Bil (µmol/L)	145.92 ± 80.11	137.64 ± 91.58	
D-Bil (µmol/L)	112.58 ± 76.45	107.74 ± 86.44	
Preoperative biliary drainage manner (PTCD/ENBD/NO)	35/2/3	43/4/5	
Biliary drainage time (d)	18.64 ± 9.75	20.31 ± 7.33	
Bile reinfusion	36	51	
ICG_15_ (%)	9.1 ± 2.7	9.2 ± 2.4	

BMI, body mass index; LsRRH, laparoscopy group; LtRRH, laparotomy group

### Preoperative assessment of tumor

Details of the preoperative assessment of the tumor are summarized in Table 2. Imaging-based assessment of tumor was emphasized on Bismuth classification, TNM stage, and vascular involvement to predetermine whether radical resection can be achieved. As shown in Table 2, the proportion of Bismuth type I in the LsRRH group was higher than in the LtRRH group. Type II and IIIb were relatively comparable, while type IIIa and IV were less in the LsRRH group (p < 0.05). In the LsRRH group, no patients had HA invasion, and 5 patients had PV involvement but not exceeding 180° of the vessel circumference. In the LtRRH group, 6 patients had HA invasion, and 13 had PV involvement. Among these 13 patients, 4 had an involvement exceeding 180° with required PV resection and reconstruction. Regarding the proportion of vascular invasion, HA and PV involvement in the LsRRH group was lower than in the LtRRH group (p < 0.05), indicating that patients with a lower level of technique requirement were enrolled selectively for LsRRH. In order to address insufficient residual liver volume following hepatectomy, 2 patients were performed preoperative portal vein embolization in LsRRH group.

**Table 2:** Preoperative assessment of tumor

	LsRRH (n = 38)	LtRRH (n = 54)	*p*Value
Bismuth classification			
Type-I (%)	7 (18.42%)	6 (11.11%)	< 0.05
Type-II (%)	3 (7.89%)	4 (7.40%)	
Type-IIIa (%)	6 (15.80%)	14 (25.92%)	< 0.05
Type-IIIb (%)	18 (47.37%)	27 (50%)	
Type-IV (%)	4 (10.53%)	11(20.37)	< 0.05
Vascular invasion in pre-reserved side	5(13.15%)	19(35.19%)	< 0.05
HA invasion	0	6(11.11%)	< 0.05
PV involvement	5(13.16%)	13(24.07%)	< 0.05

HA, hepatic artery; PV, portal vein

### Surgery strategy and intraoperative data

The surgery strategy for various Bismuth types of HCCA in our institution obeys the following principles. Bismuth type I undergoes extrahepatic bile duct resection (EBDR). Bismuth type II and IIIb undergo left hepatectomy (LHx) with total caudate lobectomy (TCL). Bismuth type IIIa undergoes right hepatectomy (RHx) with TCL. Bismuth type IV undergoes typical or extended LHx/RHx decided by the extent of tumor invasion to the left and right hepatic duct, residual liver volume, and ICG_15_%. In two patients with FLR, less than 40% received lobus quadratus resection (segment IVb+V) and TCL. Because of the high proportion of Bismuth type I, the ratio of EBDR alone in the LsRRH group was higher than in the LtRRH group (p < 0.05). And the proportion of type IIIa and type IV was lower in the LsRRH group, so the typical and extended RHx were less than in the LtRRH group (p < 0.05). All cases were combined with hilar clearance of lymph nodes, adipose, and nerve tissue containing No.12, 13, 8, and 9 lymph nodes. Wedge resection and repair were performed for preserved PV invasion but not exceeding 180°; resection and end-to-end reconstruction were carried out for those exceeding 180°. One patient in the LsRRH group scheduled for wedge resection preoperatively was transferred to segmental resection and end-to-end anastomosis when involvement exceeded 180° intraoperatively. Intraoperative frozen sections of the proximal and distal residuals of the bile duct and vessel were made to guarantee negative margins. The TCL was routinely performed in Bismuth type II, III, and type IV patients. After excluding Bismuth type I cases, the TCL ratio in the LsRRH group was higher than in the LtRRH group (p < 0.05). The mean operation time was longer for LsRRH than LtRRH (p < 0.05). The LsRRH group had less blood loss, fewer transfusion cases, and less transfusion volume (p < 0.05). The details of surgery strategy and intraoperative data are summarized in Table 3.

**Table 3:** Surgery strategy and intraoperative data

	LsRRH (n = 38)	LtRRH (n = 54)	*p*Value
Surgery approach			
EBDR alone	7(18.42%)	2(3.70%)	< 0.05
Typical or extended LHx	22(57.89%)	30(55.56%)	
Typical or extended RHx	9(23.68%)	20(37.04%)	< 0.05
TCL	29(93.55%)	33(61.11%)	< 0.05
Vessel invasion	5	19	
Anastomosis of HA	0	3(5.56%)	
HA repair	0	3(5.56%)	
Wedge resection in PV	4(10.53%)	9(16.67%)	< 0.05
PV resection and reconstruction	1(2.63%)	4(7.41%)	< 0.05
Operative time (min)	431 ± 127	357 ± 104	< 0.05
Blood loss (ml)	240 ± 130	400 ± 210	< 0.05
Blood transfusion (%)	9(23.7%)	21(38.9%)	< 0.05

LHx, left hepatic resection; RHx, right hepatic resection; EBDR, extrahepatic bile duct resection; TCL, total caudate lobectomy

### Biliary-enteric reconstruction (BER)

The biliary residuals in the hepatic segment of the couinaud was the standard for counting. Depending on different surgery strategies, the residuals range from 1 to 6, with the most residuals presented in lobus quadratus resection. The average number of the residuals in the LsRRH and LtRRH groups was 2.54 ± 1.62 and 2.47 ± 1.46, respectively (p > 0.05).

Cholangioplasty was performed to enlarge the diameter of the residuals to facilitate anastomosis and lessen the number of anastomoses. Distant biliary residuals out of plastic were subjected to choledochojejunostomy separately, and a scattered lobular duct on the liver section with a diameter less than 1 mm was ligated directly. The choledochojejunostomy ranges from 1 to 4, with a mean of 2.04 ± 1.27 and 2.57 ± 1.33 in the LsRRH and LtRRH groups, respectively (p > 0.05).

**Fig. 1:**
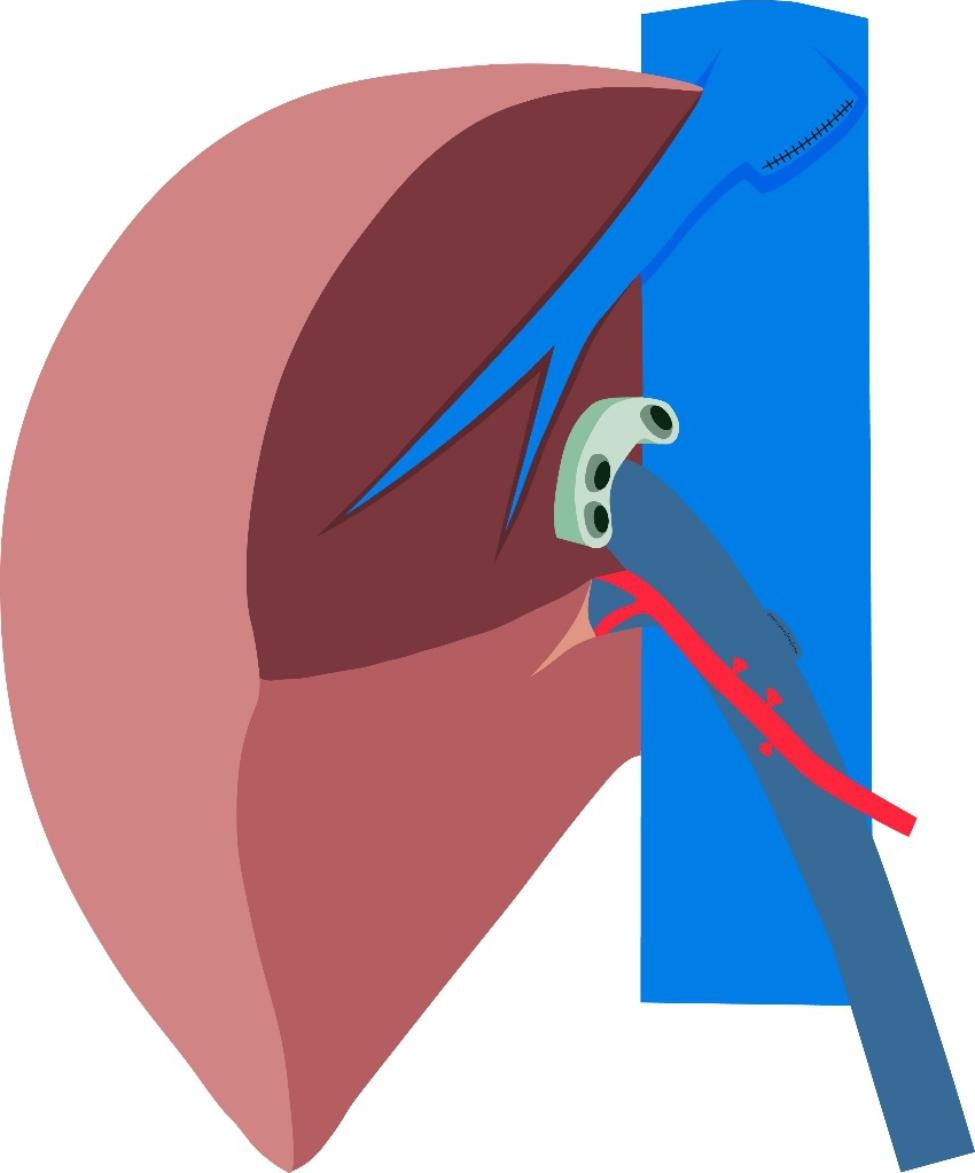
Anatomical illustration after left hemihepatectomy

**Fig. 2:**
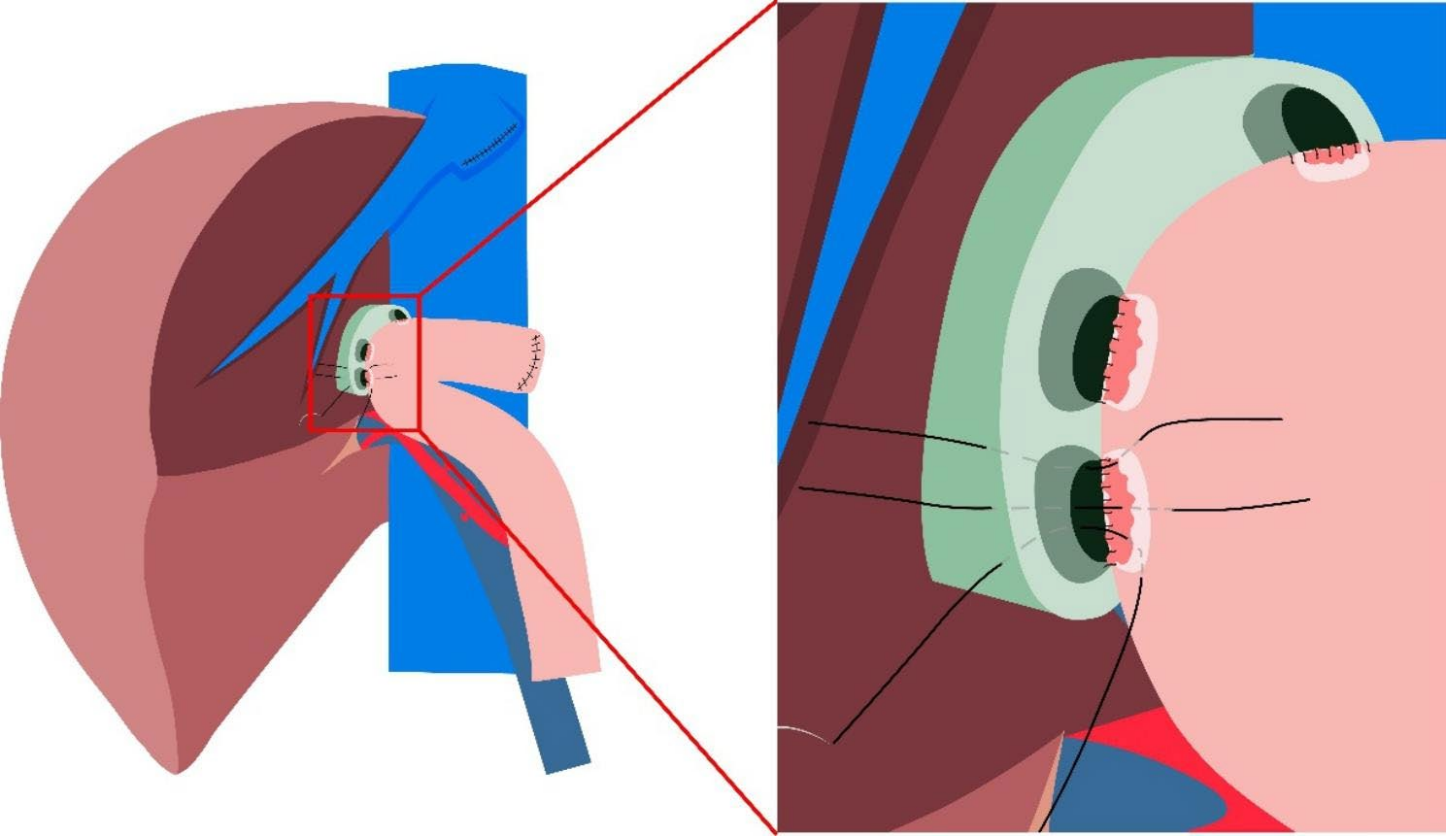
Cholangio-jejunostomy after left hemihepatectomy. Each bile duct stump is anastomosed to the jejunum separately. The figure shows that the posterior wall of the bile ducts has been anastomosed to the posterior wall of the jejunal anastomosis, and the anterior wall of the bile duct is being anastomosed to the anterior wall of the jejunal anastomosis

**Fig. 3:**
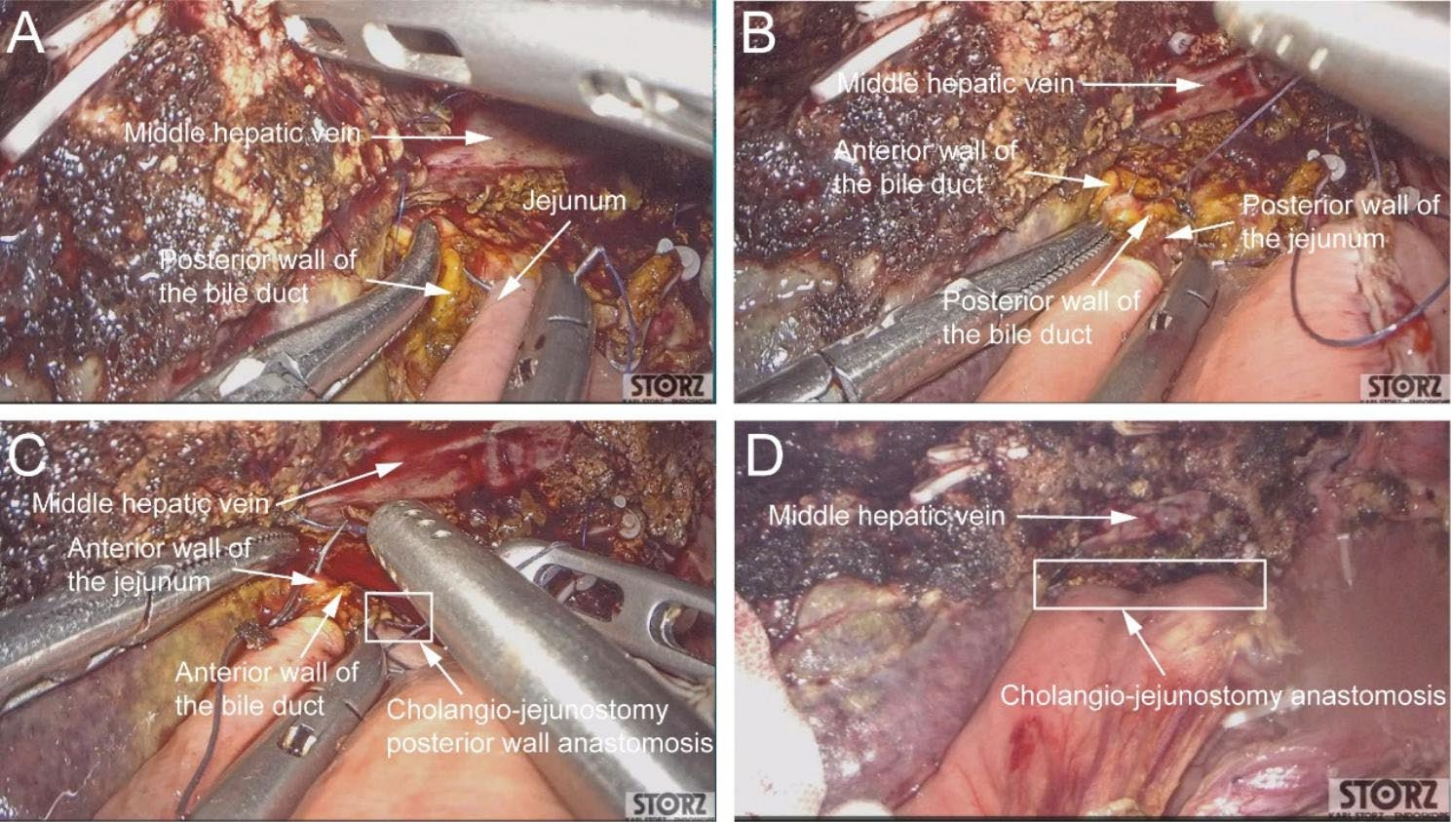
Representative photos of laparoscopic cholangio-jejunostomy after left hemihepatectomy. (**A**) Operative photograph showing the first stitch of the cholangio-jejunostomy anastomosis. (**B**) Operative photograph showing the anastomosis of the posterior wall of the bile duct with the posterior wall of the jejunum. (**C**) Operative photograph showing the anastomosis of the anterior wall of the bile duct with the anterior wall of the jejunum. (**D**) Operative photograph showing the completion of the cholangio-jejunostomy anastomosis

**Fig. 4:**
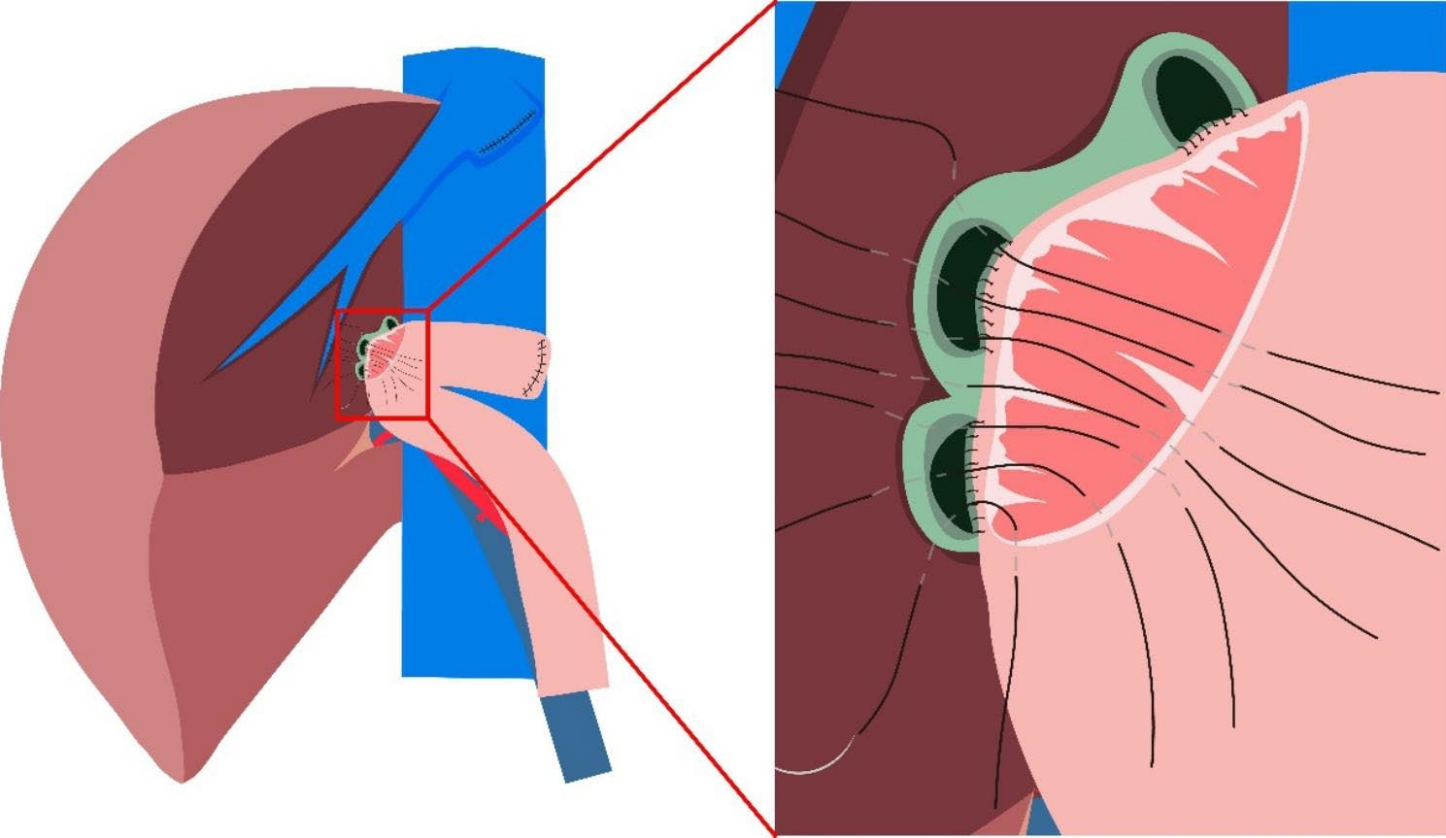
Porto-jejunostomy (also named as Kasai procedure) after left hemihepatectomy. It is used in complex cases with multiple bile duct stumps, which have small diameters and thin walls that can neither be plastic nor anastomosed separately. The figure shows that the posterior wall of bile duct stumps with surrounding tissue of Glisson pedicle have been sutured to the posterior wall of a single large anastomosis on the jejunum, and the anterior wall of the jejunal anastomosis is being anastomosed to hilar tissue

**Fig. 5:**
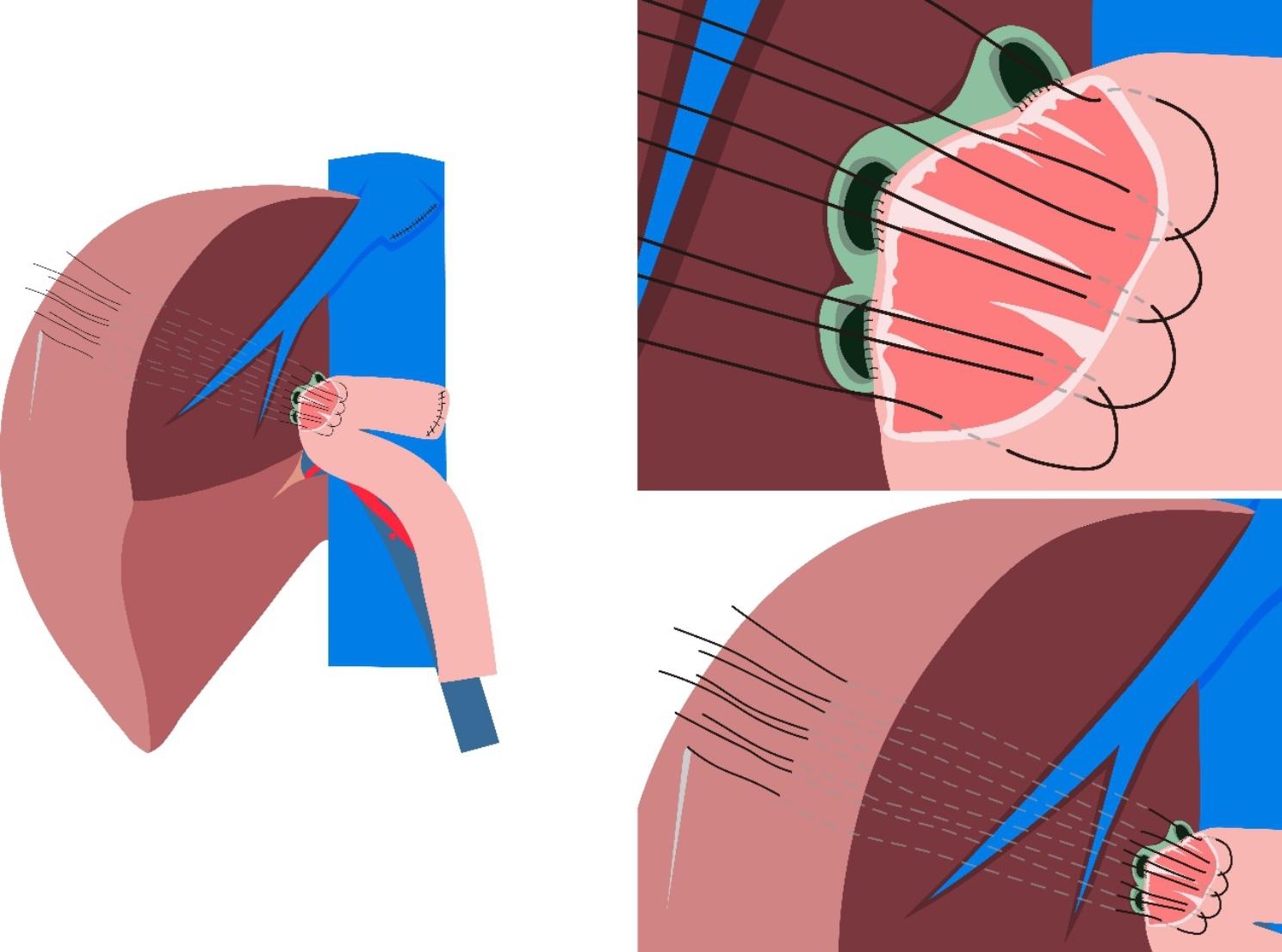
Hepato-jejunostomy after left hemihepatectomy. It is used in complex cases with multiple bile duct stumps, which have small diameters and thin walls that can neither be plastic nor anastomosed separately. And the tissue around the anterior wall of bile ducts and Glisson pedicle is insufficient for suturing. The figure shows that the posterior wall of bile duct stumps has been sutured to the posterior wall of a single large anastomosis on the jejunum, and the anterior wall of the jejunal anastomosis is intermittently sutured to the liver tissue with a straight needle

**Fig. 6:**
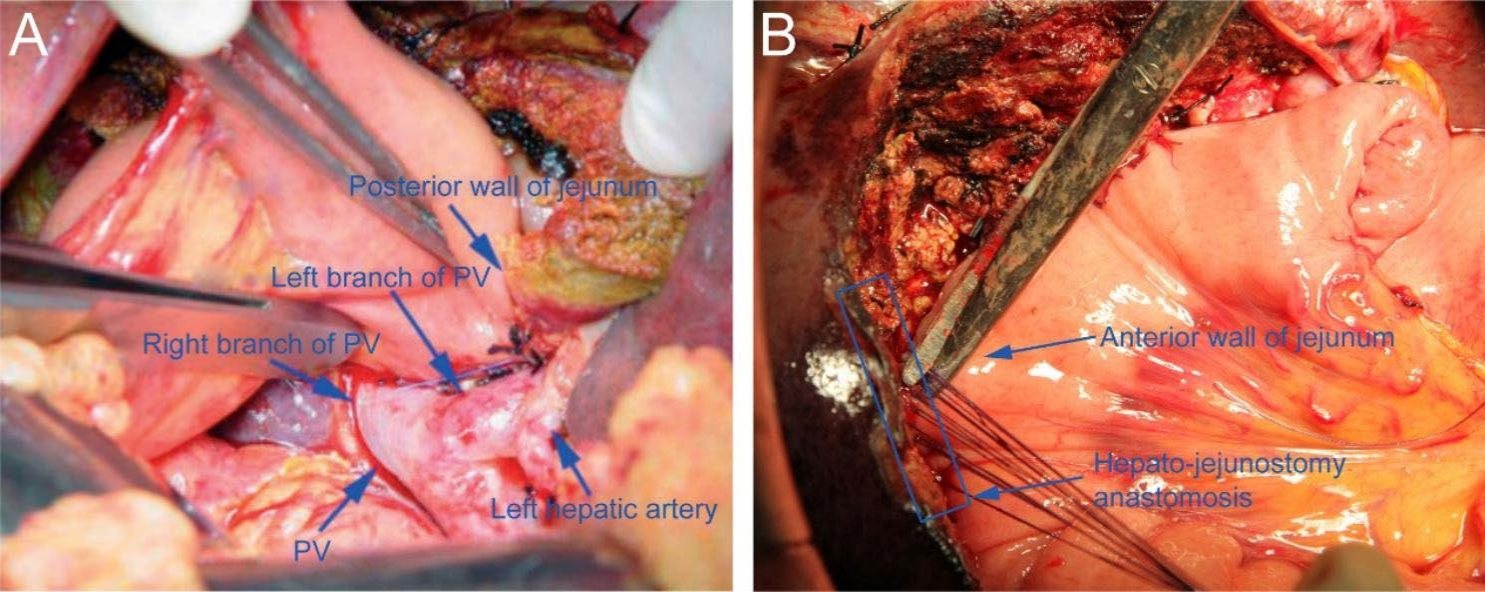
Representative photos of laparoscopic hepato-jejunostomy after left hemihepatectomy. (**A**) Operative photograph showing anastomosis involving the posterior wall of jejunum: a continuous 4/0 polypropylene suture was used to sew the seromuscular layer of the posterior wall of jejunum to the wall of the right and left branches of the portal vein [27]. (**B**) Operative photograph showing anastomosis involving the anterior wall of jejunum. The anterior wall of the jejunal anastomosis was intermittently sutured to the liver tissue and knotted on the side of the jejunum. PV, portal vein

In this article we use the example of left hemihepatectomy (Fig. 1) to show anatomical schematic diagrams of the three approaches of anastomosis. Retro-colic Roux-en-Y cholangio-jejunostomy is preferred for BER (Fig. 2). Representative photos of laparoscopic cholangio-jejunostomy before, during, and after anastomosis were showed in Fig. 3. For intrahepatic bile duct residuals diameters of 5 to 8 mm and below, end-to-side anastomoses were established with 5 − 0 absorbable monofilament sutures. And 4 − 0 was used for those with diameters above 8 mm. In complex cases with multiple stumps, small inner diameters, and thin duct walls that can neither be plastic nor anastomosed separately, a porto-jejunostomy (also named as Kasai procedure) [25] was used, which sutured the bile duct stumps with surrounding tissue of Glisson pedicle to the posterior wall of a single large anastomosis on the jejunum, and sutured the anterior wall of the jejunal anastomosis to hepatic portal tissues (Fig. 4). When the tissue around the bile ducts and Glisson pedicle is insufficient for suturing, a hepato-jejunostomy [26] is also an alternative option, in which the anterior wall of the jejunal anastomosis is intermittently sutured to the liver tissue with a straight needle (Fig. 5). In 2009, Chen Xiaoping and his team presented an operative photograph showing anastomosis involving the posterior wall of jejunum in the paper entitled extent of liver resection for hilar cholangiocarcinoma(Fig. 6A) [27]. Operative photograph showing anastomosis involving the anterior wall of jejunum was shown in Fig. 6B. We also provide schematics of the three anastomoses.

Each anastomosis was counted as 1, and those that became a whole anastomosis after cholangioplasty were also counted as 1. Therefore, cholangio-jejunostomy had 3 or 2 anastomoses in each case, depending on the number of bile duct stumps, and porto-jejunostomy and hepato-jejunostomy had 1 anastomosis. Among the 27 cholangio-jejunostom in the LsRRH group, there were 18 cases with three cholangio-jejunal anastomoses and 9 cases with two; among the 45 cholangio-jejunostom in the open surgery group, there were 23 cases with three anastomoses and 22 cases with two cholangio-jejunal anastomoses. Therefore, there were 72 cholangio-jejunal anastomoses in the LsRRH group and 113 in the LtRRH group. There were 11 cases and 2 cases of porto-jejunostomy in the LsRRH and LtRRH groups, respectively. There were also 7 cases of hepato-jejunostomy in the LtRRH group, which was not included in the LsRRH group. There was no statistical difference between the two groups in terms of anastomosis manner (*p* > 0.05).

As for the suture method, a continuous suture is suited for a long residuals with sufficient duct tissue, while an intermittent suture is done for a short residuals with scarce tissue. The discontinuous and continuous suture was performed randomly or in combination for anterior and posterior anastomosis walls depending on duct length and tissue volume. The average time of BER in the LsRRH and LtRRH groups was 65.67 ± 21.53 min and 42.5 ± 19.77 min, respectively (p < 0.05), which accounted for 15.28 ± 3.64% and 11.76 ± 2.54% of the total operation time in each group (p < 0.05). These illustrated that the time consumption ratio is significantly higher in the LsRRH group. The details of BER data are summarized in Table 4.

**Table 4:** Biliary-enteric reconstruction

	LsRRH (n = 38)	LtRRH (n = 54)	*p* Value
Number of residuals	2.54 ± 1.62	2.47 ± 1.46	
Diameter of residuals (mm)	12.37 ± 8.13	13.62 ± 4.96	
Number of anastomotic	2.04 ± 1.27	2.57 ± 1.33	
Anastomosis manner			
Cholangio-jejunostomy	72	113	
Porto-jejunostomy	11	2	
Hepato-jejunostomy	0	7	
Anastomosis time (min)	65.67 ± 21.53	42.5 ± 19.77	< 0.05
Time consumption ratio (%)	15.08 ± 3.64	11.76 ± 2.54	< 0.05
Margin status (R0/R1/R2)	37/1/0	50/3/1	

In both LsRRH and LtRRH groups, we performed intraoperative frozen-section examination to clarify whether R0 resection was achieved. 37 cases of R0 resection and 1 case of R1 resection were achieved in the LsRRH group; 50 cases of R0 resection, 3 cases of R1 resection and 1 case of R2 resection were achieved in the LtRRH group. The cases that failed to achieve R0 resection were due to the deep location of the tumor in the liver, which was difficult to reach surgically, and were further treated with chemotherapy after surgery. There was no statistical difference between the two groups in terms of resection margins (p > 0.05).

### Complications related to BER

The details of complications related to BER are summarized in Table 5. There were 15 cases of postoperative bile leakage in the two groups. 6 (15.40%) were from the LsRRH group, including 4 grade A, 2 grade B, and 0 grade C. 9 (16.67%) were in the LtRRH group, including 5 grade A, 4 grade B, and also 0 grade C. All cases in grade A were cured by unblocking abdominal drainage and without additional treatment. Of the 6 grade B, 2 patients with limited perihepatic biliary effusion were from the LsRRH group, 3 patients with restricted perihepatic biliary flow, and 1 with diffuse cholestatic peritonitis was from the LtRRH group. They were cured by B-ultrasound or CT-guided percutaneous catheter drainage. No patient with bile leakage received second surgery. Statistical analysis showed no difference in the two groups incidence and grade of bile leakage (p > 0.05).

**Table 5:** Complications related to BER

	LsRRH (n = 38)	LtRRH (n = 54)	*p*Value
Bile leakage (n, %)	6(15.79%)	9(16.67%)	
Grade (A/B/C)	4/2/0	5/4/0	
Average draining times in Grade B	2 ± 0.34	3 ± 0.42	
Peritonitis	0	1	
Limited perihepatic biliary effusion	3	4	
Average healing time (d)	14 ± 10.28	17 ± 9.73	< 0.05
Average healing time in hospital (d)	8 ± 4.61	7 ± 2.35	
Anastomotic stenosis	1(2.63%)	1(1.85%)	
Bleeding	0	0	
Death related to BER (n)	0	0	

The average healing time in LsRRH and LtRRH groups was 14 ± 10.28d and 17 ± 9.73d, respectively (p < 0.05). The prolonged healing time was due to 1 case in the LsRRH group and 2 cases in the LtRRH group whose bile leakage was delayed and discharged with a draining tube. After excluding these three cases, the average healing time was 8 ± 4.61d and 7 ± 2.35d (p > 0.05).

There was 1 case of biliary-enteric anastomotic stenosis in each group of LsRRH and LtRRH. The case in the LsRRH group was a Bismuth type IIIb who underwent LHx and TCL with two anastomoses of the right anterior and right posterior hepatic duct to the jejunum. On the 51st postoperative day, this patient presented fever with delayed jaundice, and MRCP confirmed right anterior anastomotic stenosis with stone formation. The stenosis and cholelithiasis were resolved by balloon expansion three times with percutaneous transhepatic choledochoscopy (PTCS). The case in the LtRRH group was Bismuth type IV HCCA who underwent LHx and dorsal segment of right anterior lobe and TCL with 3 anastomoses made of S5, S8, right posterior hepatic duct to the jejunum. This patient also presented fever with delayed jaundice on the 72nd postoperative day, and MRCP confirmed stenosis at S5 hepatic duct jejunostomy with stone formation. This patient was also resolved by balloon expansion twice through PTCS. There was no difference in the choice of anastomosis (cholangitis-/porto-/hepato-jejunostomy) between the LsRRH and LtRRH groups (Table 4) or in the anastomosis in which stenosis occurred.

No biliary hemorrhage occurred in either group. One patient in the LsRRH group died of hepatic failure within 72 h after surgery. Another patient in the LtRRH group died on the 17th day after being discharged due to violent vomiting resulting in hemorrhage of the cardia mucosa tear. Therefore, no patient died from complications related to BER.

## Discussion

Yu and Chen from the same institution published the first two reports on laparoscopic radical resection of hilar cholangiocarcinoma (LsRRH) in 2010 [28] and 2013 [29]. However, challenges to the safety and feasibility of LsRRH immediately arose. The main concern was to evaluate whether resectability was reliable in the absence of touch and if radicality of the tumor would be lacking due to preservation of the caudate lobe [30]. Nowadays, it is generally accepted that LsRRH is technically feasible regarding safety and radicality in high-volume institutions with highly selected patients executed with experienced surgeons. The same conclusion was reached with the data from our center, which we do not discuss here because it is outside the topic of this study. However, LsRRH remains highly controversial on minimal invasiveness because of its longer operation time than LtRRH, thus increasing trauma from more prolonged anesthesia and inflammatory response.

In the LsRRH group of our cohort, patients were relatively young, the proportion of Bismuth type I was higher while type IIIa and IV were less, and no case required revascularization. It indicated a significant selection bias of patients with better tolerance for more extended surgery and anesthesia trauma, pneumoperitoneum, and lower technique requirement were recruited for LsRRH. This bias will inevitably have a severe impact on studies related to LsRRH and lead to a biased result, especially on the radical feasibility of tumor resection and the complexity of the surgery. It should be noted that the primary purpose of this study was to investigate whether BER would be a burden in LsRRH compared to open surgery, in other words, whether BER, this surgical technique, can be applied to patients who can be operated on laparoscopically within the current conditions. And we believe this bias will be overcome by improving surgical skills. As an early attempt, our study provided a good foundation for more objective results. Such as, one patient who underwent PV resection and reconstruction because of involvement exceeding 180° was found intraoperatively and was identified without a revascularization requirement in preoperative assessment. It adjusted the selective bias to a certain degree and supplied an innovation for the BER in LsRRH.

One significant discrepancy is the anastomosis time and time consumption ratio of BER in LsRRH is longer than in LtRRH. It mainly stems from the complexity of the BER in LsRRH. First, the liver and diaphragm are lifted by pneumoperitoneum. At the same time, the stomach and intestine mesentery descended to the foot side in reverse Trendelenburg position, which increases the distance and tension between the jejunum and biliary residuals. Second, maximum bile duct and fibrous tissue in Glisson’s pedicle have been removed to achieve an R0 margin, which resulted in the biliary remnant wall being thin and embedded deeply in the liver. Third, two or multiple anastomotic stomas are required in most cases, so the previously completed anastomotic stomas occupies the space of subsequent anastomosis in LsRRH but can be ignored in LtRRH.

To alleviate the impediment of these disadvantages of laparoscopy on BER, save anastomosis time, and reduce postoperative complications, we summarized a few principles to facilitate BER in LsRRH from our experience. One is that a variety of anastomosis manners (choledochojejunostomy without or with cholangioplasty, portojejunostomy, hepato-jejunostomy) should be reasonably and comprehensively applied in on cases. The other is that two suture methods (continuous and interrupted suture) can be utilized independently or jointly in one or different biliary residuals. The third is the “Easy First” principle for multiple anastomoses. Specifically, right anterior anastomosis should be after right posterior in LHx, left external anastomoses should be after left internal in RHx, and right anastomose should be before left in central hepatectomy. Fourth, the surgeon in charge should have accomplished at least 30 cases of biliary reconstruction in laparoscopic pancreaticoduodenectomy (LPD). We believe that as the number of cases increases and operators become more experienced, the operating time will be reduced accordingly. The increase in the number of participants is the basis for technological innovation. As the number of medical centers performing LsRRH rises, more and more operators will summarize their experience and improve their surgical approach. The improvements in technology will then bring about a major change in LsRRH, which will further reduce the operative time. Multidisciplinary collaboration is also a potential way to reduce the time to LsRRH, such as improved suture materials that reduce suture time and visualization techniques that allow laparoscopic manipulation with a three-dimensional view closer to that of open surgery, thus facilitating manipulation.

Regarding postoperative bile leakage, the incidences were equal in the LsRRH and LtRRH groups, which indicated that the quality of BER in laparoscopy could reach that level in laparotomy. Despite the disadvantages mentioned above in laparoscopy, the magnification effect of laparoscope reduces the suture space and results in a more delicate needle gauge and stitch interval. In the LsRRH group, one patient died within 72 h postoperatively from liver failure unrelated to the BER. A patient presented right anterior hepatic anastomosis stenosis followed by secondary bile stones and recurrent cholangitis. The potential causes may be complicated and challenging to be identified clearly. Using an ultrasonic scalpel to transect the bile duct is more likely to induce scar formation, making the increased probability of postoperative anastomotic stenosis may be a compelling factor. However, variation was not found between LsRRH group and LtRRH group about anastomosis (cholangitis-/porto-/Hepato-jejunum anastomosis) (Table 4).

The biliary enteric anastomotic stoma is an outflow channel for purer bile without a mixture of pancreatic and gastric-intestinal fluids. Therefore, the impact is relatively minor compared to the bile leakage in pancreaticoduodenectomy, which is mixed with pancreatic fluid. So, the necessary treatment is adequate drainage to prevent abdominal infection. We experience that once a bile leakage is diagnosed, the patient should reduce turning and movement to avoid bile dispersing and spreading before abdominal adhesion is completed. Repeated multiple punctures for drainage are mandatory. If bile leakage cannot be limited, biliary peritonitis worsens, or the patient develops some systemic infection symptoms, such as persistent fever, abdominal distention, and intestinal paralysis, a second operation with appropriate management is required.

In summary, there is a significant selection bias of patients in this study which mainly affects tumor resection rather than BER. Laparoscopic BER has both advantages and disadvantages. In LsRRH, BER is technically feasible, and the quality of anastomosis is equal to that of open surgery evaluated from the postoperative bile leakage and other complications. However, BER takes longer and a more significant proportion of total operation time, indicating that it has higher technical requirements and is a critical rate-limiting factor affecting the minimal invasiveness of LsRRH.

## Data Availability

All data generated or analysed during this study are included in this published article.
